# Impact of National Volume-Based Procurement on the Procurement Volumes and Spending for Antiviral Medications of Hepatitis B Virus

**DOI:** 10.3389/fphar.2022.842944

**Published:** 2022-06-06

**Authors:** Jing Yuan, Z. Kevin Lu, Xiaomo Xiong, Tai-Ying Lee, Huang Huang, Bin Jiang

**Affiliations:** ^1^ Department of Clinical Pharmacy & Pharmacy Administration, School of Pharmacy, Fudan University, Shanghai, China; ^2^ University of South Carolina College of Pharmacy, Columbia, SC, United States; ^3^ Department of Public Policy, School of Government, Peking University, Beijing, China; ^4^ Department of Pharmacy Administration & Clinical Pharmacy, School of Pharmaceutical Sciences, Peking University, Beijing, China

**Keywords:** NVBP, HBV, affordability, procurement, access to care, ITS

## Abstract

**Introduction:** Although persistent inhibition of HBV replication by antiviral therapy has shown to slow disease progression, cost-related access barriers to these essential medicines are becoming salient. The national volume-based procurement (NVBP) was piloted in China and led to substantial reduction in the list price of prescription drugs. To examine the impact of NVBP on selected antiviral medication costs per defined daily dose (DDD), procurement volumes, and spending.

**Methods:** We employed an interrupted time series design to examine changes in cost per defined daily dose (DDD), procurement volumes, and spending for NVBP bid-winning antiviral medications (tenofovir disoproxil fumarate and entecavir) in 11 pilot cities from 2017 to 2020. Procurement transaction data were obtained from 9,454 hospitals in the Chinese Hospital Pharmaceutical Audit (CHPA) database. In the secondary analysis, the control group comprised two non-NVBP drugs (adefovir and lamivudine) procured in 11 cities not exposed to the NVBP.

**Results:** Cost per DDD of the two hepatitis B virus (HBV) antiviral medications reduced by CNY1.598 (*p* = 0.002) immediately following the implementation of NVBP, dropping from an average cost of CNY16.483 per DDD at baseline to CNY6.420 at the end of the observation period. NVBP implementation resulted in a substantial reduction in daily costs of antivirals and an increase in monthly procurement volumes by 6.674 million DDDs (*p* = 0.017), while monthly spending was reduced by CNY138.26 million (*p* = 0.002). In the secondary ITS analysis with a control group, the average cost per DDD of the NVBP bid-winning antivirals declined by CNY4.537 (*p* < 0.001), monthly procurement volumes increased by 7.209 million DDDs (*p* = 0.002), and monthly spending dropped by CNY138.83 million (*p* < 0.001).

**Conclusion:** Volume-based procurement piloted in China may be effective for reducing price and total expenditures and improving drug utilization, which is especially important for HBV patients who need constant access to antiviral therapies.

## Highlights

What is already known?

1) Potential public health benefits in reducing transmission of chronic hepatitis B virus (HBV) could be achieved through wider use of antiviral therapy and expansion of HBV treatment eligibility.
2) National volume-based procurement (NVBP), the novel pooled procurement to negotiate drug prices with manufacturers by using collective bargaining power, has successfully reduced medication price and improved medication affordability in China.

What are the new findings?

1) Using national procurement transaction data, the NVBP had an impact on reducing drug prices and improving the utilization of antivirals for chronic HBV.
2) Beyond the increased use of antiviral drugs for HBV, the spending on NVBP bid-winning medications decreased substantially over time.

What do the new findings imply?

1) NVBP piloted in China may serve as an effective tool for reducing prescription prices.
2) The sustainable effect of NVBP in improving access to medications provides invaluable insights for decision-makers navigating ways to improve patient access to care.

## Introduction

Aimed at reducing global HBV incidence by 90% and mortality by 65%, the UN’s Sustainable Development Goals have set ambitious targets, projecting these to be achieved by 2030 (McNaughton et al., 2021). Generally, effective vaccines and supportive care are recommended for preventing and managing of Hepatitis B. As complete eradication of HBV cannot be achieved by modern HBV therapy, people with HBV may need to depend on their medications for the rest of their lives (Vlachogiannakos and Papatheodoridis, 2018). Persistent inhibition of HBV replication by antiviral therapy has been shown to be able to prevent chronic HBV‐induced progressive fibrosis, cirrhosis, and HCC (Lampertico et al., 2015; Papatheodoridis et al., 2015), achieving the ultimate goal of improved survival and quality of life for most HBV patients (Vlachogiannakos and Papatheodoridis, 2018). Through wider use of antiviral therapy and expansion of HBV treatment eligibility, potential public health benefits in reducing transmission may be achieved as suggested by McNaughton AL and colleagues (McNaughton et al., 2021). However, antiviral therapy can be costly, resulting in increased economic burden and limited access to such essential medications. In low-to-middle-income countries (LMICs) such as China, where access to medicines is largely influenced by the drug price, this can become an issue.

As one of the most important measures of China’s health care system reform and universal health coverage program, the national volume-based procurement (NVBP) was implemented in December 2018. Specifically, the NVBP is a novel pooled procurement for negotiating drug prices with manufacturers by using collective bargaining power, and was piloted in the “4+7” cities, comprising four municipalities (Beijing, Shanghai, Tianjin, and Chongqing) and seven key cities in other provinces (Xi’an, Dalian, Guangzhou, Chengdu, Shenzhen, and Xiamen) (Yuan et al., 2021). The NVBP is administrated by the Joint Procurement Office (JPO), which is a coalition of representatives from local drug procurement agencies established under the State Council. The NVBP has taken several steps to reduce prices and increase the affordability of higher quality medicines, including ensuring bioequivalence and therapeutic efficacy of generic drugs, achieving volume-price linkage to improve negotiation power, promoting drug supply chain efficiency, encouraging generic substitution and rational use of medication, and reducing capital and marketing costs. (Yuan et al., 2021).

NVBP primarily aims to lower drug prices while maintaining the supply of pharmaceuticals, and substantial cuts in drug prices have been reported upon its implementation (Tang et al., 2019; Yue, 2019). Increased procurement prices were attributed to the “winner-takes-all” principle applied in procurement (Jiang et al., 2020). Furthermore, based on the data on previous pharmaceutical reforms in China, the effects of lowering prescription drug prices might become insignificant in the long run (He et al., 2018). A number of studies have found that the implementation of the NVBP was associated with overall decreased drug prices and expenditures, along with an increased overall quality level of drug use (Chen et al., 2020; Chen et al., 2021; Wang et al., 2021). A previous study by our team also found that the implementation of the NVBP improved the affordability of the drugs procured (Yuan et al., 2021). In addition, several studies have also assessed the effect of NVBP on some specialized drugs, including antibiotics, antihypertensive drugs, and nucleic acid drugs. These studies found that the NVBP might have a positive effect on different types of drugs (Yang et al., 2021a; Yang et al., 2021b; Wen et al., 2021). However, the study on the nucleos(t)ide analogs only included the data in one of the pilot cities of the NVBP for a relatively short period of time (Wen et al., 2021). Hence, the impact of NVBP on drug prices, total expenditures, and utilization of antiviral therapy in all seven pilot cities over a longer period of time remains unclear.

To better understand the impact of NVBP, this study aimed to examine the policy’s impact on selected antiviral medication costs per defined daily dose (DDD), procurement volumes, and spending. Two antiviral medications, tenofovir disoproxil fumarate (TDF) and entecavir, were included in the NVBP. TDF is mainly indicated for the treatment of human immunodeficiency virus type 1 (HIV-1) infection in combination with other antiretroviral agents, but it is mainly used for patients with HBV, given the extremely low prevalence of HIV in China11. It was estimated that the prevalence of HIV was approximately 0.5% in a high-risk population in China (Zhang et al., 2013).

## Methods

### Overview of National Volume-Based Procurement in China

To achieve universal health coverage (UHC), China has launched radical health reforms to improve access to care and, particularly, lowering prescription drug prices. As part of the pharmaceutical reform, the NVBP pilot program was launched in March 2019 in “4+7” cities. Procurement included originators or generics passing the generic consistency evaluation (GCE). A total of 25 drugs won the bidding in the “4+7” pilot program, including two HBV antivirals (TDF and entecavir). A careful evaluation of the “4+7” piloted policy showed that NVBP expanded to the remaining provinces of the nation in December 2019. The elements of the NVBP include ensuring bioequivalence and therapeutic efficacy of generic drugs, achieving volume-price linkage to improve negotiation power, promoting drug supply chain efficiency, encouraging generic substitution and rational use of medication, and reducing capital and marketing costs. The implementation details of each element can be found in our previously published article (Yuan et al., 2021).

### Data Source

National data from 1 January 2017 to 30 September 2020 were obtained from the Chinese Hospital Pharmaceutical Audit (CHPA) database (Diao et al., 2019). CHPA provides a national estimate based on drug purchase data of 9,454 hospitals sampled from all hospitals with over 100 beds in four municipalities, 27 provinces, and 255 major and county-level cities in China. According to the 2020 Statistical Bulletin on the Development of China’s Health and Wellness, the number of hospitals with ≥100 beds was 14,148 at the end of 2020, indicating that the coverage rate of the hospitals with (≥100 beds) in the CHPA might be more than half (Commission, 2020). As antivirals are specialty prescription drugs in China, most HBV patients fill their prescriptions in hospital pharmacies rather than in community pharmacies. We extracted monthly aggregated purchases of antivirals with detailed information on generic name, brand name, the Anatomical Therapeutic Chemical code, manufacturer, strength, package specification, total volumes, and costs of purchase. (Diao et al., 2019). Additionally, a standardized dosing unit was defined by CHPA. Only data from the public hospital were included in the analysis as NVBP was implemented exclusively in public hospitals.

### Study Design

To evaluate NVBP impact, we employed an interrupted time series (ITS) design with three segments divided by two intervention points. The implementation of the NVBP in 11 pilot cities was the intervention for this study. Specifically, initial procurement of two antivirals (TDF and entecavir) was implemented between March and April 2019, and NVBP was expanded between November and December 2019. Therefore, the 43-months time series was divided into three segments; the pre-NVBP period was defined as the time period between January 2017 and March 2019, the “4+7” pilot period was between April and November 2019, and the expansion period was between December 2019 and September 2020. ITS analysis, one of the strongest quasi-experimental designs, is commonly used to determine whether a new program or policy is associated with specific outcomes. This is particularly useful when conducting a randomized clinical trial. By collecting multiple time points before and after intervention, detecting an underlying secular trend in the pre- and post-intervention phases is possible. This study followed the methodological and reporting guidelines for interrupted time-series analyses proposed by (Jandoc et al., 2015).

### Patient and Public Involvement

As the hospital procurement data were used in this study, the patient and public were not involved in the design, conduct, and dissemination of this research.

### Definition of Variables

Outcomes included cost per DDD, procurement volumes, and spending for each of the four antivirals marketed in China (i.e., TDF, entecavir, adefovir, and lamivudine). Cost per DDD was used as a surrogate measure for unit price paid by public hospitals. Cost per DDD was calculated for each of the antivirals by dividing procurement spending and the volume of a given drug. As the actual daily dose may vary between patients over time, DDD, defined as the assumed average maintenance daily dose for a drug used for adults, was used as a proxy for daily dose (Organization, 2021; Zhang et al., 2021). DDDs were obtained from the ATC Index published by the World Health Organization (WHO) Collaborating Centre for Drug Statistics Methodology (WHO, 2021). Procurement volumes comprised the total monthly DDDs for each antiviral of interest. Procurement spending was calculated to reflect the expenditures paid by hospitals.

### Statistical Analysis

#### Primary Analysis

We performed segmented linear regression analyses with repeated measures for each outcome separately (Wagner et al., 2002; Koskinen et al., 2015). The following equation was used for the regression:

$$Y_{t}=\beta_{0}+\beta_{1}T_{t}+\beta_{2}X_{t}+\beta_{3}X_{t}T_{t}.$$ (1)

Here, Y_t_ is the dependent variable with quarterly values (cost per DDD, procurement volumes, and procurement spending); X_t_ is a dummy variable representing the implementation of NVBP; X is defined as 0 for the pre-intervention period, 1 for the period between the implementation of NVBP pilot and before NVBP expansion, and 2 for the period after the NVBP expansion; T_t_ is the number of months since the start of the study; and X_t_T_t_ is an interaction term between time and intervention.

Segmented linear regression model tested whether the time point when the NVBP was implemented (March 2019) was associated with immediate change (the level or the regression coefficient β_1_) at the time of intervention, a change in the trajectory trend (the slope or the regression coefficient β_2_) of outcomes beginning at intervention, or both.

### Secondary Analysis

As the intervention effect may be disentangled from other underlying or unobserved influences (Jandoc et al., 2015), we also performed ITS analysis with a control group unexposed to the intervention. To estimate secular changes in utilization of antivirals outside pilot cities, we selected another 11 cities unexposed to the NVBP as control cities. As NVBP impact might be diffused between provinces, we chose two HBV antivirals (adefovir and lamivudine) deemed to be exchangeable with TDF and entecavir. These control cities were chosen as they have similar population sizes, geographic locations, and economical aggregates compared to the pilot cities. Selected control cities included Changchun, Changsha, Hefei, Hangzhou, Kunming, Lanzhou, Nanjing, Ningbo, Qingdao, Wuhan, and Zhenjiang as follows:

$$Y_{t}=\beta_{0}+\beta_{1}T_{t}+\beta_{2}X_{t}+\beta_{3}X_{t}T_{t}+\beta_{4}Z+\beta_{5}ZT_{t}+\beta_{6}ZX_{t}+\beta_{7}ZX_{t}T_{t}+\varepsilon_{t}$$ (2)

Here, Z is a dummy variable denoting the intervention assignment (intervention = 1, control = 0). Segmented linear regression model tested whether the time point at which NVBP was implemented (March 2019) was associated with immediate change (the level or the regression coefficient β_6_) at the time of intervention, a change in the trajectory trend (the slope or the regression coefficient β_7_) of outcomes (i.e., daily spending, utilization, and total expenditures) starting at the intervention, or both.

The results of the parameter estimate from the models and visual representations of time series graphs are provided. The Durbin-Watson statistic was used to test the autocorrelation of errors (Durbin and Watson, 1951). Cochrane-Orcutt autoregression procedure was performed to adjust for first-order correlated errors (Applied linear regression models, 2004). Mixed effect models were used to account for clustering by hospital. Dataset management was conducted using the SAS 9.4. All statistical analyses were performed using Stata version 16 (StataCorp). Statistical significance was set at *p* < 0.05.

## Results

For the “4+7” cities, a total of 6,246 and 11,301 procurement transactions for TDF and adefovir, respectively, were included in the primary ITS analysis. For control cities, a total of 12,685 and 5,617 procurement transactions for entecavir and lamivudine, respectively, were included in the secondary ITS analysis. Supplementary Appendix Table A1 lists the selected HBV antivirals. Supplementary Appendix Table A2 presents the Durbin-Watson statistics.

### Impacts on Cost per Defined Daily Dose

**FIGURE 1:**
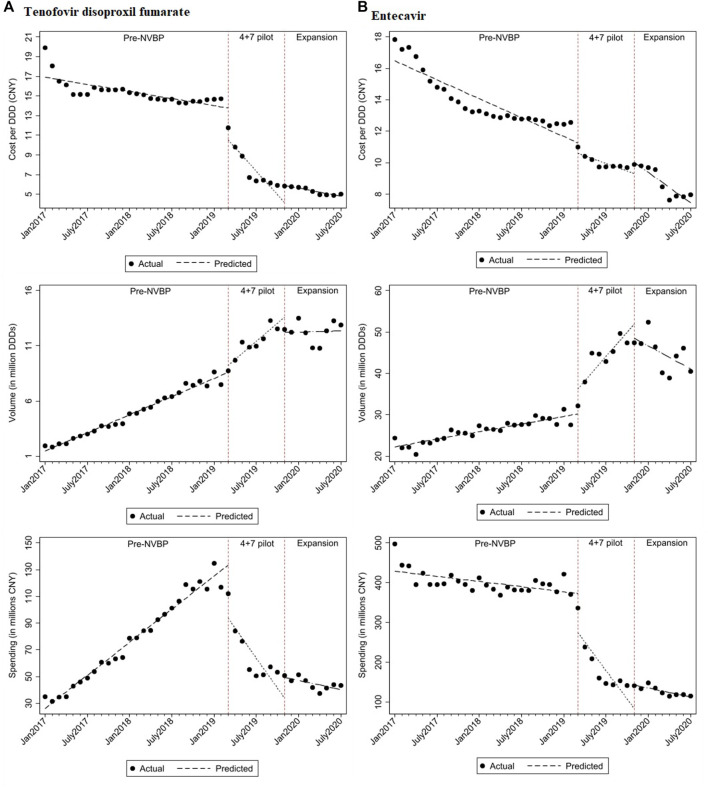
The observed and predicted **(A)** TDF and **(B)** entecavir from aggregated ITS analysis*. * The predicted values were obtained from aggregated ITS analysis for TDF and entecavir procured in "4+7” cities.

**FIGURE 2:**
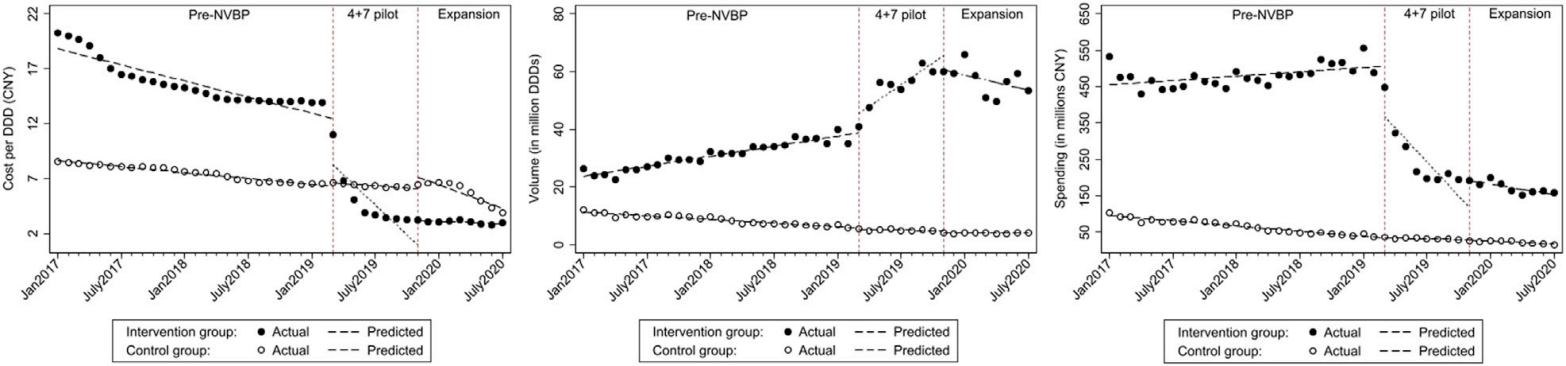
The observed and predicted **(A)** cost per DDD **(B)** procurement volume **(C)** procurement spending from secondary ITS analysis with a control group*. * The predicted values were obtained from ITS with a control groups, the intervention group included TDF and entecavir procured in "4+7” cities; control group included adefovir and lamivudine procured in control cities.

In the aggregated controlled ITS analysis, the cost per DDD of NVBP antivirals in the “4+7” cities decreased abruptly and significantly in March 2019 (Figure 1A). Implementing the NVBP, the cost per DDD of the two HBV antivirals had dropped by CNY1.598 (*p* = 0.002) on average from an average baseline cost of CNY16.483 per DDD just before the intervention (Table 1). Downward trend continued (trend change of CNY0.239 per month; *p* = 0.015). With initiation of NVBP expansion (or 8 months after NVBP), cost per DDD of antivirals had dropped by CNY1.005 (*p* = 0.023) on average, and average cost per DDD reached CNY6.420 at the end of the observation period. Our secondary analysis showed that when including the control group, average cost per DDD of the NVBP antivirals procured in the “4+7” cities had declined by CNY4.537 (*p* < 0.001) compared with the non-NVBP antivirals procured in the control cities (Table 2). NVBP expansion had insignificant impact on the cost per DDD of the intervention group compared to the control group (Figure 2).

**TABLE 1:** Changes in levels and trends of HBV antivirals on cost per DDD, procurement volume, and procurement spending for antivirals in “4+7″ pilot cities^a^.

Variables	Aggregated antivirals^a^			TDF			Entecavir		
	Estimate (95% CI)		*p*-Value	Estimate (95% CI)		*p*-Value	Estimate (95% CI)		*p*-Value
Cost per DDD (RMB)									
Slope change prior to intervention	−0.166	(−0.24–−0.09)	<0.001	−0.120	(−0.21–−0.03)	0.007	−0.200	(−0.26–−0.14)	<0.001
***Effect of NVBP procurement*** ()									
Level change	−1.598	(−2.58–−0.61)	0.002	−3.244	(−4.70–−1.79)	<0.001	−0.666	(−1.50–0.16)	0.113
Trend change	−0.239	(−0.43–−0.05)	0.015	−0.676	(−1.00–−0.35)	<0.001	0.040	(−0.07–0.15)	0.485
***Effect of NVBP expansion*** (Wang et al., 2019)									
Level change	1.005	(0.15–1.86)	0.023	1.733	(0.19–3.28)	0.029	0.708	(0.13–1.28)	0.017
Trend change	0.165	(−0.01–0.34)	0.061	0.658	(0.35–0.96)	<0.001	−0.158	(−0.28–−0.03)	0.015
**Hospital monthly volume (in million DDDs)**									
Slope change prior to intervention	0.580	(0.49–0.67)	<0.001	0.274	(0.25–0.29)	<0.001	0.306	(0.23–0.38)	<0.001
***Effect of NVBP procurement*** ()									
Level change	6.674	(1.26–12.09)	0.017	0.641	(−0.09–1.37)	0.082	6.033	(1.28–10.79)	0.014
Trend change	1.923	(0.81–3.04)	0.001	0.271	(0.11–0.43)	0.001	1.652	(0.68–2.62)	0.001
***Effect of NVBP expansion*** (Wang et al., 2019)									
Level change	−4.815	(−10.46–0.83)	0.092	−1.372	(−2.54–−0.20)	0.023	−3.444	(−8.10–1.21)	0.142
Trend change	−3.402	(−4.78–−2.02)	<0.001	−0.529	(−0.77–−0.29)	<0.001	−2.873	(−4.05–−1.69)	<0.001
**Hospital monthly spending (in million RMB)**									
Slope change prior to intervention	1.972	(−0.03–3.97)	0.054	4.129	(3.82–4.43)	<0.001	−2.157	(−3.95–−0.36)	0.020
***Effect of NVBP procurement*** ()									
Level change	−138.26	(−223.4–−53.12)	0.002	−39.864	(−61.03–−18.70)	<0.001	−98.398	(−163.17–−33.62)	0.004
Trend change	−33.220	(−50.92–−15.52)	0.001	−11.589	(−15.84–−7.33)	<0.001	−21.631	(−35.13–−8.14)	0.002
***Effect of NVBP expansion*** (Wang et al., 2019)									
Level change	74.176	(−8.73–157.09)	0.078	15.452	(−5.76–36.67)	0.148	58.724	(−3.10–120.55)	0.062
Trend change	26.342	(8.55–44.13)	0.005	6.327	(1.88–10.77)	0.006	20.015	(6.61–33.42)	0.004

a Aggregated ITS, included TDF, and entecavir. 1. NVBP, was introduced in “4+7” pilot cities in March 2019. 2. NVBP, was expanded to the nation in November 2019.

**TABLE 2:** Secondary analysis of ITS with a control group on cost per DDD, procurement volume, and procurement spending for antivirals^a^.

Variables	Cost per DDD (RMB)			Hospital Monthly Volume (in Million DDDs)			Hospital Monthly Spending (in Million RMB)		
	Estimate (95% CI)		*p*-Value	Estimate (95% CI)		*p*-Value	Estimate (95% CI)		*p*-Value
*Preintervention period*									
Control level (intercept) on Jan2017	8.607	(8.47–8.74)	<0.001	11.359	(10.78–11.94)	<0.001	95.782	(90.98–100.58)	<0.001
Control monthly trend (slope) prior to intervention	−0.090	(−0.10–−0.08)	<0.001	−0.213	(−0.24–−0.18)	<0.001	−2.368	(−2.66–−2.08)	<0.001
Difference of NVBP vs. control in level	10.206	(8.85–11.56)	<0.001	12.362	(10.93–13.79)	<0.001	359.302	(329.8–388.8)	<0.001
Difference of NVBP vs. control in trend change	−0.155	(−0.24–−0.07)	0.001	0.793	(0.70–0.89)	<0.001	4.339	(2.39–6.29)	<0.001
***Effects related to “4+7” NVBP*** ()									
Difference of NVBP vs. control in level change immediately following intervention initiation	−4.537	(−6.94–−2.13)	<0.001	7.209	(2.68–11.74)	0.002	−138.83	(−221.5–−56.1)	0.001
Difference of NVBP vs. control in trend change immediately following intervention initiation	−0.689	(−1.24–−0.14)	0.015	1.761	(0.79–2.73)	0.001	−34.930	(−52.34–−17.52)	<0.001
***Effects related to NVBP expansion*** (Wang et al., 2019)									
Difference of NVBP vs. control in level change immediately following intervention initiation	1.164	(−1.25–3.58)	0.340	−4.008	(−8.82–0.80)	0.101	74.718	(0.32–149.11)	0.049
Difference of NVBP vs. control in trend change immediately following intervention initiation	1.156	(0.61–1.70)	<0.001	−3.448	(−4.67–−2.23)	<0.001	27.139	(9.74–44.54)	0.003

a To test the effects of NVBP, in ITS, with a control group, the intervention group included TDF, and entecavir procured in “4+7” cities; the comparison group included adefovir and lamivudine procured in control cities. 1. NVBP, was introduced in “4+7” pilot cities in March 2019. 2. NVBP, was expanded to the nation in November 2019.

**FIGURE 3:**
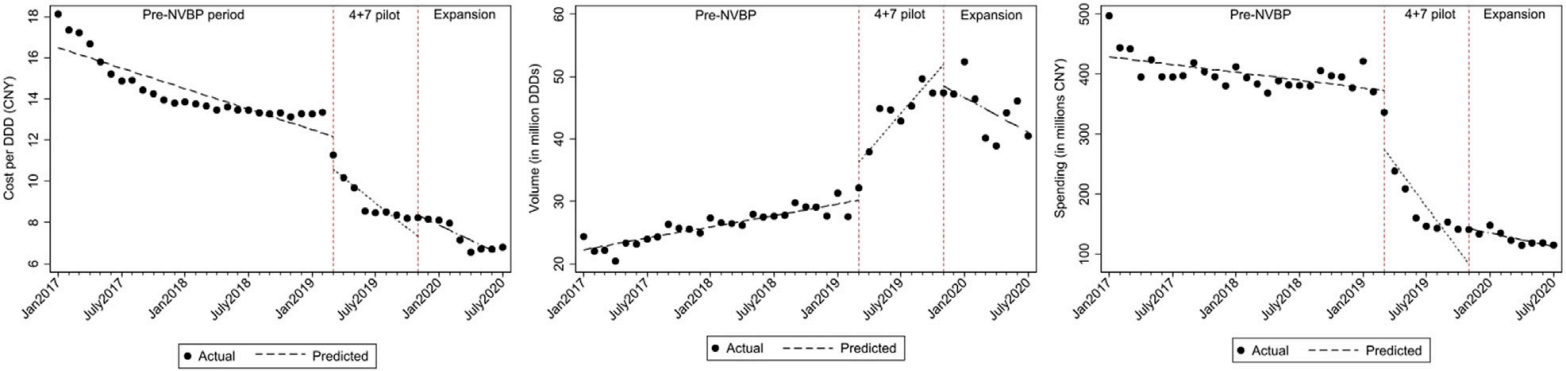
The observed and predicted outcomes values from aggregated ITS analysis for **(A)** cost per DDD **(B)** procurement volume **(C)** procurement spending.

For individual antivirals, both TDF and entecavir had a reduction in cost per DDD (Figure 3). For TDF procured in the “4+7” cities, the baseline cost of TDF was CNY16.900 and dropped significantly by CNY3.244 with the implementation of NVBP (Table 2; *p* < 0.001). At the end of the observation period, the cost of TDF reached CNY4.795 per DDD. In the secondary analysis, average cost per DDD of TDF declined significantly by CNY5.462 (Supplementary Appendix Table A3; *p* < 0.001) compared with non-NVBP antivirals procured in the control cities. For entecavir, cost per DDD dropped by CNY0.666 on average after launching NVBP but without reaching statistical significance (Table 2; *p* = 0.113). Cost of entecavir was reduced from CNY16.477 at baseline to CNY7.472 at the end of the observation period. In the secondary analysis, with the implementation of NVBP, cost of entecavir dropped significantly by CNY4.447 (Supplementary Appendix Table A3; *p* = 0.001), compared with the control group.

### Impact on Procurement Volumes

Figure 1 shows that with the reduction in cost per DDD in “4+7” cities associated with the NVBP, procurement volumes increased substantially at the time of NVBP implementation. In aggregated controlled ITS analysis, immediately after implementing the NVBP, the monthly procurement volumes of two HBV antivirals had increased by 6.674 million DDDs (*p* = 0.017) on average from an average baseline volume of 23.720 million DDDs just before intervention (Table 1). The upward trend continued (trend change of 1.923 million DDDs per month; *p* = 0.001), and monthly procurement volume reached 53.481 million DDDs at the end of the observation period. In the secondary ITS analysis, compared to the control group, monthly procurement volumes increased by 7.209 million DDDs (Table 2; *p* = 0.002).

Given the reduced daily cost for NVBP antivirals in “4+7” cities, both TDF and entecavir had increased procurement volumes (Figure 3). With the implementation of the NVBP, monthly procurement volumes of TDF had increased by 0.641 million DDDs (Table 1; *p* = 0.082), rising from 1.463 million DDDs at baseline to 12.342 million DDDs at the end of the study period. In secondary ITS analysis, the TDF procurement volumes had increased by 1.176 million DDDs immediately after the introduction of NVBP (Supplementary Appendix Table A3; *p* = 0.007), compared to non-NVBP antivirals procured in control cities. For entecavir, with the implementation of NVBP, procurement volumes increased by 6.033 million DDDs (Table 2; *p* = 0.014). Entecavir procurement volumes increased from 22.258 million DDDs at the baseline to 41.140 million DDDs at the end of the observation period. In the secondary analysis, entecavir volumes increased significantly by 6.568 million DDDs (Supplementary Appendix Table A3; *p* = 0.007), compared with the control group.

### Impact on Procurement Spending

Even though the consumption volumes of antiviral drugs increased substantially, the daily costs and medication spending of the two NVBP antivirals decreased over time (Figure 1). In the aggregated controlled ITS analysis, monthly spending of the two HBV antivirals had dropped by CNY138.26 million on average immediately after implementation the NVBP (Table 1; *p* = 0.002), from an average spending of CNY455.084 million at baseline to CNY153.020 million at the end of the study period. In secondary ITS analysis, the monthly procurement spending of the two NVBP antivirals decreased by CNY138.83 million (Table 2; *p* = 0.001).

Figure 3 shows that the monthly procurement spending for both TDF and entecavir dropped after the implementation of NVBP over time. Accordingly, the monthly procurement spending of TDF had decreased by CNY39.864 million (Table 1; *p* < 0.001), with a downward trend of CNY11.589 million per month. At the end of the study period, the monthly spending reached CNY40.297 million in “4+7” cities. In secondary ITS analysis, procurement spending of TDF increased by CNY1.710 million immediately after the introduction of NVBP (Supplementary Appendix Table A3; *p* < 0.001), compared to non-NVBP antivirals procured in control cities. For entecavir, with the implementation of NVBP, procurement spending had decreased by CNY98.398 million (Table 2; *p* = 0.004). In our secondary analysis, entecavir spending increased significantly by CNY98.966 million DDDs (Supplementary Appendix Table A3; *p* = 0.003), compared with the control group.

## Discussion

In this study, we assessed the impact of NVBP on the utilization and expenditure of antiviral agents used for HBV infection. Our findings fulfilled the hypothesis that NVBP decreased the daily spending (or price) of antivirals, increasing the utilization (or demand) of medication treatment. Considering substantial price cuts for both TDF and entecavir, a drastic decrease in the total expenditures can be observed. This suggests the potential effect of NVBP on controlling total expenditures while improving affordability and patient access to previously costly antiviral agents.

Our findings are consistent with previous studies. Wen et al. found that in Shenzhen, the NVBP pilot program was associated with reduced DDDs and improved accessibility of nucleos(t)ide analogs. (Wen et al., 2021). Our study further investigated the impact of the NVBP on antiviral drugs in all pilot cities with a longer period of study time. We found that the NVBP extension also had a positive effect. Wang et al. found that the NVBP is conducive to generic substitution. There is a similar effect in our study. (Wang et al., 2021). As the generic version of TDF was approved in 2017, we observed gradually expanded utilization of TDF over time. For adefovir and lamivudine (two drugs not included in the NVBP), the utilization remained stable, suggesting a significant effect of NVBP on increasing antiviral therapy for HBV patients. In addition, some studies have found that NVBP had a positive effect on antihypertensive and antibiotics. (Yang et al., 2021a; Yang et al., 2021b). However, there is no relevant research on the differences in the effects for different types of drugs, which may require more follow-up research.

NVBP explored the potential ways to reduce high drug prices, which is one of the greatest global public health issues faced by many nations. Prior to using NVBP, antiviral treatment may cause significant financial burden and distress because of its unaffordability to a large proportion of hepatitis patients in China. (Diao et al., 2019). Using NVBP as an innovative strategy for drug price regulation, bid-winning drugs—specifically TDF—demonstrated its favorable profiles by exhibiting 94% price cuts and significant change in the total expenditures on TDF by 8.8 times from 1.506 million on 2017 Q1 to 13.148 million on 2020 Q3. As the generic version of TDF was approved in 2017, we observed gradually expanded utilization of TDF over time. For adefovir and lamivudine (two drugs not included in the NVBP), the utilization remained stable, suggesting a significant effect of NVBP on increasing antiviral therapy for HBV patients. Additionally, the prices for brand-name drugs also decreased slightly. This might be explained by increased competition in the drug market for antiviral therapy, where unselected antiviral drugs are actively lowering their prices, ultimately expanding patient access to more treatment options.

Price negotiations with manufacturers based on volume purchases have long been considered effective at reducing drug prices (Gaffney and Lexchin, 2018). For instance, a price cut of generic drugs has been achieved through the Partnership for Supply Chain Management (PFSCM), which provided procurement services to the United States government (Tren et al., 2009; van Valen et al., 2018). Another example of NVBP was found in Italy, where an immense budgetary concern among patients needing sofosbuvir was addressed by introducing nationwide budget thresholds and price-volume agreements. Consequently, reduced prices and expanded access to treatment by more patients were achieved (Messori, 2016).

Antiviral medications are essential for the successful treatment of hepatitis. Implementing NVBP may help reduce disease transmission and achieve UN goals (McNaughton et al., 2021). Individuals with HBV may experience both personal and societal impacts, including anxiety, financial burden and instability, discrimination, and stigma (Tu et al., 2020). Timely access to antiviral therapy is key in regulating disease progression and improving quality of life, while current therapies are expensive and generally require life-long treatment (Vedio et al., 2017; Alter et al., 2018; Revill et al., 2019). In low-to-middle-income countries (LMIC) with high prevalence of HBsAg, the main challenges are the availability and cost of antiviral therapy (Subic and Zoulim, 2018). Improving access to this essential therapy for a large population is a complex public health challenge requiring multilateral collaborations among pharmaceutical companies, government agencies, and healthcare providers. The novel implementation of NVBP in China demonstrated its effectivity in streamlining access to essential medicines for vulnerable populations. Most recently, healthcare systems have been moving toward services emphasizing value-based purchasing and patient-centered care (Chee et al., 2016). Similarly, future procurement in China should consider the value of healthcare products. Clinicians and patients should actively engage in the procurement process to maximize optimal outcomes, achieve shared goals, and make informed decisions according to the value of the drugs (VanLare and Conway, 2012).

As part of the health care reforms implemented in China in recent years, NVBP has demonstrated its role in promoting the rationality of medical use. At present, the expansion of NVBP is still in progress, and its effect may also help more patients to improve their health equity and quality of life. In any case, most of the pilot cities of NVBP are economically developed cities, and how implementing the policy in remote or rural areas still needs a lot of effort. At the same time, in these remote places, it is unknown whether the effect will be as same as the effect in metropolitan areas. In addition, the types of drugs included in NVBP are still limited, and how to expand the policy to more drugs also needs to be considered. In drug price negotiations, pharmacoeconomics may play a supporting role. The cost-effectiveness analysis for the drug itself and the budget impact analysis for insurance funding can both play their roles and help determine the negotiated price more rationally and scientifically.

Our study bears several strengths. First, we employed big data from 9,454 hospitals nationwide and interrupted time series studies. These have helped us substantially in understanding the price change of antiviral treatment for HBV as drug pricing is not transparent in China before NVBP. Additionally, this is also the first study to evaluate changes in the utilization of hepatitis medications before and after the implementation of NVBP. However, our study contains several limitations. First, the data collected in this study may not be generalizable to some developed countries using value-based purchasing in their respective health systems and may not reflect the most desirable measures that will be adopted by providers in various institutions. Besides, private hospitals and retail pharmacies were encouraged to participate but not mandatory for the NVBP. However, private hospital beds account for about a quarter of China’s total (Commission, 2020), therefor, future studies needs to investigate the effect of the NVBP on private hospitals, especially comparing those with or without the NVBP. Additionally, our data cannot be used to make inferences about individual-level outcomes as patient factors; responses to therapy may vary. Third, even though we chose control cities with similar social and economic characteristics, huge differences in procurement volumes and spending between pilot and control cities were found. Also, the secular change might have an effect on the results. In any case, the control cities with regression method could minimize the bias caused by the other factors. Fourth, NVBP programs are not adequately developed and designed to encompass all patent hepatitis medications requiring price reduction, better affordability, and easy access for patients (Penfold and Zhang, 2013). Future researchers should increase focus on the transitions from volume-based to value-based models in China. Nevertheless, the implementation and application of such measures could profoundly influence drug price, expenditure, utilization, and access to antiviral therapy in China (Daub et al., 2020).

## Conclusion

Volume-based procurement can be a very powerful tool for reducing drug prices and lowering total expenditures on expensive drugs, which may further enhance patients’ access to these drugs. This is especially important when patients require persistent therapy for committable diseases, such as HBV. Future studies are warranted to better understand the long-term impact of NVBP on access to medicine.

## Data Availability

The raw data supporting the conclusions of this article will be made available by the authors, without undue reservation.
