# Supplementary material for: Impact of National Volume-Based Procurement on the Procurement Volumes and Spending for Antiviral Medications of Hepatitis B Virus
Source: Front Pharmacol. 2022 Jun 6;13:842944. doi: 10.3389/fphar.2022.842944 (PMC9207460; doi:10.3389/fphar.2022.842944)
Supplement: Supplementary file 1 [file DataSheet1.docx]

**Appendix**

**Appendix Table 1. Description of antiviral medications for HBV**

| **Generic Names** | **Indication** | **WHO-ATC Code** | **DDD** |
| --- | --- | --- | --- |
| Entecavir | Treatment of chronic hepatitis B virus (HBV) infection | J05AF10 | 0.5 mg |
| Tenofovir disoproxil fumarate | Treatment of chronic hepatitis B virus (HBV) and Human Immunodeficiency Virus (HIV) infection | J05AF07 | 0.245 g |
| Adefovir | Treatment of chronic hepatitis B | J05AF08 | 10 mg |
| Lamivudine | Treatment of chronic hepatitis B virus (HBV) infection | J05AF05 | 0.3 g |
| WHO-ATC, World Health Organization Anatomical Therapeutic Chemical classification; DDD, defined daily dose. | | | |

**Appendix Table 2. Durbin–Watson test results**

|  | **Hospital monthly volume**  **(in million DDDs)** | | | **Hospital monthly spending**  **(in million RMB)** | | | | **Cost per DDD (RMB)** | | |
| --- | --- | --- | --- | --- | --- | --- | --- | --- | --- | --- |
|  | **Lag (0)** | **Lag (1)** | **Lag (2)** | **Lag (0)** | **Lag (1)** | **Lag (2)** | **Lag (0)** | | **Lag (1)** | **Lag (2)** |
| **Aggregated NVBP** | | | | | | | | | | |
| Durbin–Watson d statistic | 0.602 |  |  | 0.196 |  |  | 0.210 | |  |  |
| Durbin’s alternative test | 31.734 (P<.001) | 0.207 (P=0.652) | 0.197 (P=0.822) | 174.165 (P<.001) | 0.094 (P=0.761) | 3.830 (P=0.031) | 154.264 (P<.001) | | 0.237 (P=0.629) | 0.257 (P=0.775) |
| **TDF** | | | | | | | | | | |
| Durbin–Watson d statistic | 0.698 |  |  | 0.0825 |  |  | 0.162 | |  |  |
| Durbin’s alternative test | 27.757 (P<.001) | 0.000 (P=0.990) | 0.045 (P=0.957) | 320.871 (P<.001) | 1.830 (P=0.185) | 7.396 (P=0.002) | 210.036 (P<.001) | | 3.365 (P=0.075) | 2.488 (P=0.098) |
| **Entecavir** | | | | | | | | | | |
| Durbin–Watson d statistic | 0.461 |  |  | 0.212 |  |  | 0.331 | |  |  |
| Durbin’s alternative test | 27.757 (P<.001) | 0.731 (P=0.398) | 0.359 (P=0.701) | 159.241 (P<.001) | 0.607 (P=0.441) | 4.155 (P=0.024) | 0.240 (P<.001) | | 0.607 (P=0.627) | 2.361 (P=0.109) |
| **Adefovir** | | | | | | | | | | |
| Durbin–Watson d statistic | 0.892 |  |  | 0.523 |  |  | 0.377 | |  |  |
| Durbin’s alternative test | 27.757 (P<.001) | 0.394  (P=0.534) | 0.275 (P=0.761) | 16.208 (P<.001) | 0.097 (P=0.758) | 2.947 (P=0.066) | 60.274 (P<.001) | | 3.096 (P=0.087) | 1.611 (P=0.214) |
| **Lamivudine** | | | | | | | | | | |
| Durbin–Watson d statistic | 1.150 |  |  | 0.574 |  |  | 0.168 | |  |  |
| Durbin’s alternative test | 7.045 (P=0.011) | 1.520  (P=0.226) | 1.076 (P=0.352) | 25.091 (P<.001) | 3.219 (P=0.081) | 2.212 (P=0.125) | 130.058 (P<.001) | | 2.226 (P=0.144) | 1.291 (P=0.288) |

**Appendix Table 3. Secondary analysis of ITS with a control group on cost per DDD, procurement volume, and procurement spending for individual antivirals***

| **Measures** | **TDF** | | | **Entecavir** | | |
| --- | --- | --- | --- | --- | --- | --- |
|  | **Estimate (95% CI)** | | **P-value** | **Estimate (95% CI)** | | **P-value** |
| **Cost per DDD (RMB)** | | | | | | |
| ***Preintervention period*** | | | | | | |
| Control level (intercept) on Jan2017 | 8.607 | (8.49 – 8.72) | <.001 | 8.607 | (8.49 – 8.72) | <.001 |
| Control monthly trend (slope) prior to intervention | -0.090 | (-0.10 – -0.08) | <.001 | -0.090 | (-0.10 – -0.08) | <.001 |
| Difference of NVBP vs. control in level | 8.295 | (7.79 – 8.80) | <.001 | 10.449 | (9.23 – 11.66) | <.001 |
| Difference of NVBP vs. control in trend change | 0.026 | (0.00 – 0.06) | 0.096 | -0.186 | (-0.26 – -0.11) | <.001 |
| ***Effects related to "4+7" NVBP*** | | | | | | |
| Difference of NVBP vs control in level change immediately following intervention initiation | -5.462 | (-8.20 – -2.73) | <.001 | -4.447 | (-7.09 – -1.80) | 0.001 |
| Difference of NVBP vs control in trend change immediately following intervention initiation | -1.028 | (-1.63 – -0.43) | 0.001 | -0.617 | (-1.19 – -0.05) | 0.034 |
| ***Effects related to NVBP expansion*** | | | | | | |
| Difference of NVBP vs control in level change immediately following intervention initiation | 1.417 | (-1.27 – 4.11) | 0.297 | 1.100 | (-1.27 – 3.47) | 0.358 |
| Difference of NVBP vs control in trend change immediately following intervention initiation | 1.257 | (0.64 – 1.87) | <.001 | 1.127 | (0.56 – 1.70) | <.001 |
| **Volume (in million DDDs)** | | | | | | |
| ***Preintervention period*** | | | | | | |
| Control level (intercept) on Jan2017 | 11.359 | (10.83 – 11.89) | <.001 | 11.359 | (10.83 – 11.89) | <.001 |
| Control monthly trend (slope) prior to intervention | -0.213 | (-0.24 – -0.18) | <.001 | -0.213 | (-0.24 – -0.18) | <.001 |
| Difference of NVBP vs. control in level | -9.896 | (-10.47 – -9.32) | <.001 | 10.899 | (9.62 – 12.17) | <.001 |
| Difference of NVBP vs. control in trend change | 0.487 | (0.45 – 0.52) | <.001 | 0.519 | (0.44 – 0.60) | <.001 |
| ***Effects related to "4+7" NVBP*** | | | | | | |
| Difference of NVBP vs control in level change immediately following intervention initiation | 1.176 | (0.32 – 2.03) | 0.007 | 6.568 | (1.87 – 11.27) | 0.007 |
| Difference of NVBP vs control in trend change immediately following intervention initiation | 0.109 | (-0.06 – 0.27) | 0.190 | 1.490 | (0.54 – 2.44) | 0.003 |
| ***Effects related to NVBP expansion*** | | | | | | |
| Difference of NVBP vs control in level change immediately following intervention initiation | -0.565 | (-1.75 – 0.62) | 0.346 | -2.637 | (-7.22 – 1.95) | 0.256 |
| Difference of NVBP vs control in trend change immediately following intervention initiation | -0.575 | (-0.82 – -0.33) | <.001 | -2.919 | (-4.08 – -1.76) | <.001 |
| **Spending (in million RMB)** | | | | | | |
| ***Preintervention period*** | | | | | | |
| Control level (intercept) on Jan2017 | 95.782 | (90.92 – 100.64) | <.001 | 95.782 | (90.92 – 100.64) | <.001 |
| Control monthly trend (slope) prior to intervention | -2.368 | (-2.66 – -2.08) | <.001 | -2.368 | (-2.66 – -2.08) | <.001 |
| Difference of NVBP vs. control in level | -69.68 | (-75.82 – -63.54) | <.001 | 333.20 | (304.80 – 361.60) | <.001 |
| Difference of NVBP vs. control in trend change | 6.497 | (6.08 – 6.91) | <.001 | 0.210 | (-1.58 – 2.00) | 0.815 |
| ***Effects related to "4+7" NVBP*** | | | | | | |
| Difference of NVBP vs control in level change immediately following intervention initiation | -40.432 | (-61.63 – -19.24) | <.001 | -98.966 | (-162.79 – -35.14) | 0.003 |
| Difference of NVBP vs control in trend change immediately following intervention initiation | -13.299 | (-17.51 – -9.09) | <.001 | -23.341 | (-36.62 – -10.06) | 0.001 |
| ***Effects related to NVBP expansion*** | | | | | | |
| Difference of NVBP vs control in level change immediately following intervention initiation | 15.994 | (-5.12 – 37.11) | 0.136 | 59.265 | (-1.62 – 120.15) | 0.056 |
| Difference of NVBP vs control in trend change immediately following intervention initiation | 7.123 | (2.71 – 11.54) | 0.002 | 20.812 | (7.62 – 34.01) | 0.002 |
| * To test the effects of NVBP in ITS with a control group, the intervention group included TDF and entecavir procured in "4+7" cities; the comparison group included adefovir and lamivudine procured in control cities. 1. NVBP was introduced in "4+7" pilot cities in March 2019. 2. NVBP was expanded to the nation in November 2019. | | | | | | |

**Appendix Table 4. Changes in levels and trends of non-NVBP antivirals on cost per DDD, procurement volume, and procurement spending for antivirals in control cities***

| **Variables** | **Aggregated^*^** | | | **Adefovir** | | | **Lamivudine** | | |  |
| --- | --- | --- | --- | --- | --- | --- | --- | --- | --- | --- |
|  | **Estimate (95% CI)** | | **P-value** | **Estimate (95% CI)** | | **P value** | **Estimate (95% CI)** | | **P value** |  |
| **Cost per DDD (RMB)** | | | | | | | | | | |
| Slope change prior to intervention | -0.076 | (-0.08 – -0.07) | <.001 | -0.065 | (-0.09 – -0.04) | <.001 | -0.164 | (-0.21 – -0.12) | <.001 |  |
| ***Effect of NVBP procurement^1^*** |  |  |  |  |  |  |  |  |  |  |
| Level change | 0.507 | (0.35 – 0.66) | <.001 | 0.202 | (-0.13 – 0.53) | 0.226 | 1.063 | (0.29 – 1.84) | 0.009 |  |
| Trend change | 0.029 | (0.02 – 0.04) | <.001 | 0.052 | (0.01 – 0.09) | 0.011 | 0.096 | (0.04 – 0.16) | 0.003 |  |
| ***Effect of NVBP expansion^2^*** |  |  |  |  |  |  |  |  |  |  |
| Level change | 0.292 | (-0.17 – 0.75) | 0.205 | 0.157 | (-0.12 – 0.44) | 0.263 | 0.257 | (-0.22 – 0.73) | 0.278 |  |
| Trend change | -0.158 | (-0.24 – -0.08) | <.001 | -0.160 | (-0.21 – -0.11) | <.001 | 0.197 | (0.13 – 0.26) | <.001 |  |
| **Hospital monthly volume (in million DDDs)** | | | | | | | | | | |
| Slope change prior to intervention | -0.213 | (-0.21 – -0.18) | <.001 | -0.142 | (-0.169 – -0.115) | <.001 | -0.061 | (-0.07 – -0.05) | <.001 |  |
| ***Effect of NVBP procurement^1^*** |  |  |  |  |  |  |  |  |  |  |
| Level change | -0.535 | (-1.01 – -0.06) | 0.027 | -0.170 | (-0.494 – 0.155) | 0.297 | -0.241 | (-0.44 – -0.04) | 0.020 |  |
| Trend change | 0.162 | (0.10 – 0.22) | <.001 | 0.038 | (-0.003 – 0.080) | 0.069 | 0.010 | (-0.02 – 0.04) | 0.549 |  |
| ***Effect of NVBP expansion^2^*** |  |  |  |  |  |  |  |  |  |  |
| Level change | -0.807 | (-1.11 – 0.51) | <.001 | -0.090 | (-0.372 – 0.192) | 0.523 | 0.033 | (-0.24 – 0.31) | 0.810 |  |
| Trend change | 0.046 | (-0.01 – 0.10) | 0.125 | 0.141 | (0.033 – 0.250) | 0.012 | 0.011 | (-0.05 – 0.07) | 0.687 |  |
| **Hospital monthly spending (in million RMB)** | | | | | | | | | | |
| Slope change prior to intervention | -2.368 | (-2.66 – -2.07) | <.001 | -1.407 | (-1.73 – -1.09) | <.001 | -0.982 | (-1.11 – -0.86) | <.001 |  |
| ***Effect of NVBP procurement^1^*** |  |  |  |  |  |  |  |  |  |  |
| Level change | 0.568 | (-3.49 – 4.63) | 0.778 | 2.285 | (-1.80 – 6.37) | 0.264 | -1.613 | (-3.24 – 0.01) | 0.052 |  |
| Trend change | 1.710 | (1.25 – 2.17) | <.001 | 0.801 | (0.41 – 1.19) | <.001 | 0.449 | (0.28 – 0.61) | <.001 |  |
| ***Effect of NVBP expansion^2^*** |  |  |  |  |  |  |  |  |  |  |
| Level change | -0.541 | (-3.87 – 2.79) | 0.744 | 1.301 | (-0.90 – 3.50) | 0.239 | 0.377 | (-0.45 – 1.20) | 0.360 |  |
| Trend change | -0.797 | (-1.43 – -0.17) | 0.015 | -0.521 | (-1.01 – -0.03) | 0.039 | 0.244 | (0.11 – 0.38) | 0.001 |  |
| * non-NVBP antivirals included adefovir and lamivudine. 1. NVBP was introduced in "4+7" pilot cities in March 2019. 2. NVBP was expanded to the nation in November 2019. | | | | | | | | | | |

**Appendix Table 5. Results of the additional analysis for excluding adefovir in control cities.**

| **Variables** | | **Cost per DDD (RMB)** | | | **Hospital monthly volume** | | | **Hospital monthly spending** | | |  |
| --- | --- | --- | --- | --- | --- | --- | --- | --- | --- | --- | --- |
|  |  |  |  |  | **(in million DDDs)** | | | **(in million RMB)** | | |  |
|  |  | **Estimate (95% CI)** | | **P-value** | **Estimate (95% CI)** | | **P-value** | **Estimate (95% CI)** | | **P-value** |  |
| ***Preintervention period*** | | | | | | | | | | | |
|  | Control level (intercept) on Jan2017 | 14.935 | (14.730-15.140) | <.001 | 26.719 | (25.304-28.134) | <.001 | 402.372 | (380.722-424.021) | <.001 |  |
|  | Control monthly trend (slope) prior to intervention | -0.129 | (-0.145--0.112) | <.001 | 0.324 | (0.232-0.415) | <.001 | 0.418 | (-0.842-1.678) | 0.511 |  |
|  | Difference of NVBP vs. control in level | 2.945 | (1.907-3.983) | <.001 | 0.560 | (-1.469-2.589) | 0.584 | 92.120 | (52.931-131.309) | <.001 |  |
|  | Difference of NVBP vs. control in trend change | -0.085 | (-0.152--0.018) | 0.013 | 0.195 | (0.062-0.327) | 0.005 | 0.572 | (-1.825-2.969) | 0.636 |  |
| ***Effects related to "4+7" NVBP^1^*** | | | | | | | | | | | |
|  | Difference of NVBP vs control in level change immediately following intervention initiation | -4.678 | (-7.253--2.103) | 0.001 | 9.132 | (3.416-14.848) | 0.002 | -121.390 | (-208.300--34.480) | 0.007 |  |
|  | Difference of NVBP vs control in trend change immediately following intervention initiation | -0.579 | (-1.136--0.022) | 0.042 | 1.600 | (0.463-2.736) | 0.006 | -32.099 | (-49.805--14.393) | 0.001 |  |
| ***Effects related to NVBP expansion^2^*** | | | | | | | | | | | |
|  | Difference of NVBP vs control in level change immediately following intervention initiation | 3.604 | (0.650-6.558) | 0.017 | 9.132 | (3.416-14.848) | 0.002 | 135.948 | (45.771-226.124) | 0.004 |  |
|  | Difference of NVBP vs control in trend change immediately following intervention initiation | 1.537 | (0.876-2.198) | <.001 | 1.600 | (0.463-2.736) | 0.006 | 59.997 | (40.452-79.542) | <.001 |  |
| * To test the effects of NVBP in ITS with a control group, the intervention group included TDF and entecavir procured in "4+7" cities; the comparison group included adefovir and lamivudine procured in control cities. | | | | | | | | | | | |
| 1. NVBP was introduced in "4+7" pilot cities in March 2019. | | | | | | | | | | | |
| 2. NVBP was expanded to the nation in November 2019. | | | | | | | | | | | |

**Appendix Figure 1. The observed and predicted (A) cost per DDD, (B) procurement volume, (C) procurement spending for aggregated non-NVBP antivirals***


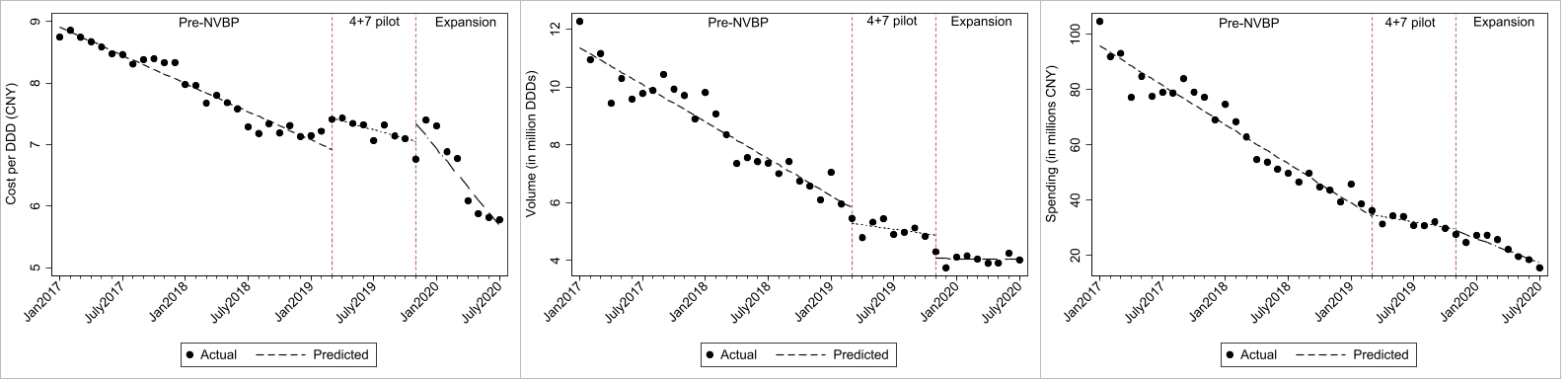


* The predicted values were obtained from aggregated ITS analysis for adefovir and lamivudine procured in control cities.

Appendix Figure 2. The observed and predicted outcomes values from aggregated ITS analysis for (A) adefovir and (B) lamivudine


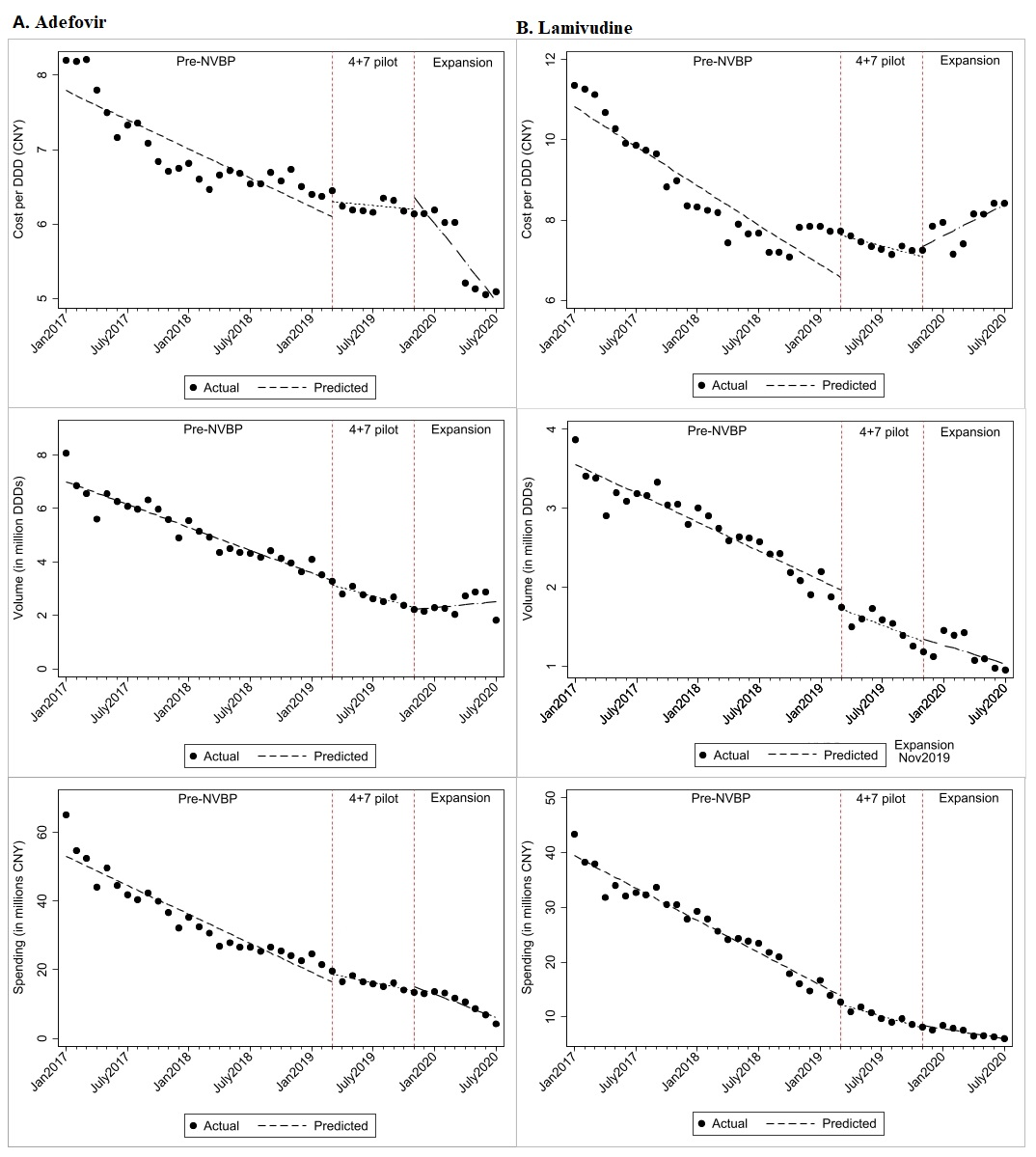


**Figure 3. The observed and predicted (A) cost per DDD, (B) procurement volume, (C) procurement spending for TDF from secondary ITS analysis with a control group***


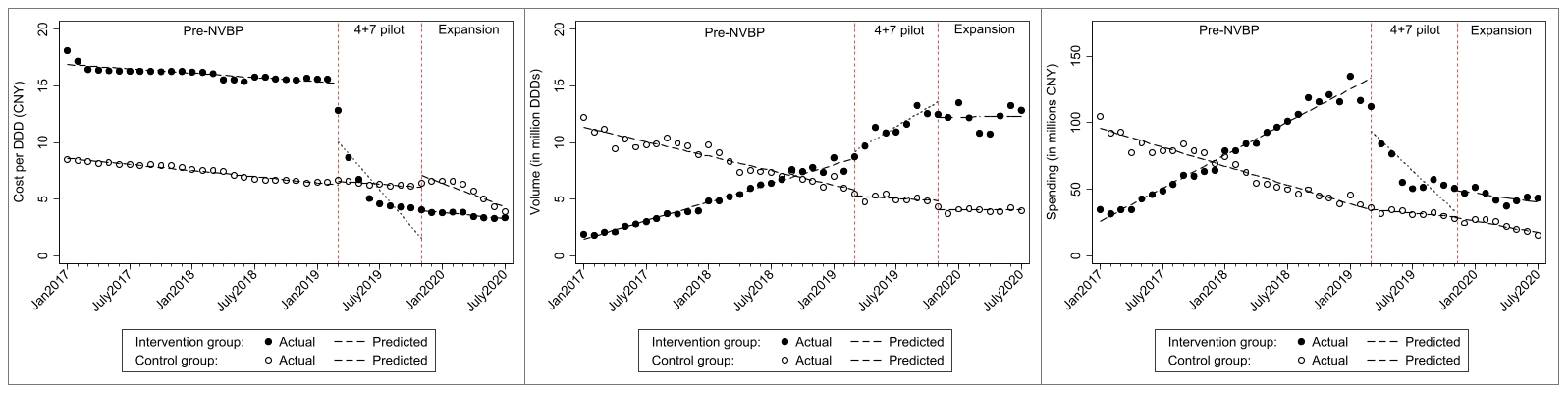


* The predicted values were obtained from ITS with a control group, the intervention group included TDF procured in "4+7" cities; the control group included adefovir and lamivudine procured in control cities.

**Figure 4. The observed and predicted (A) cost per DDD, (B) procurement volume, (C) procurement spending for lamivudine from secondary ITS analysis with a control group***


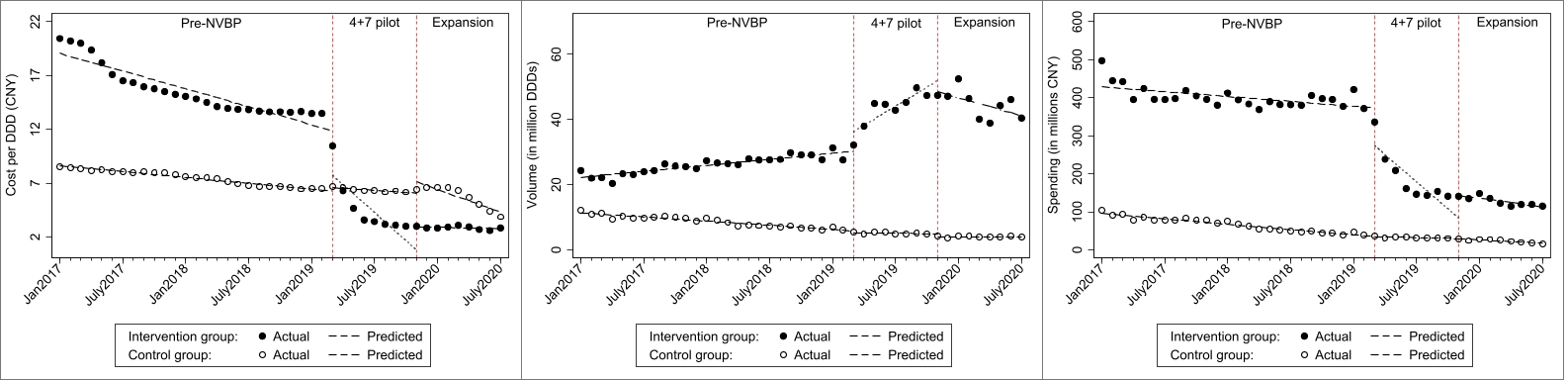


* The predicted values were obtained from ITS with a control groups, the intervention group included lamivudine procured in "4+7" cities; control group included adefovir and lamivudine procured in control cities.
